# The Effect of Cementitious Macrocapsule Addition on the Hardened Properties of Concrete with Different Packing Structures

**DOI:** 10.3390/ma18061302

**Published:** 2025-03-15

**Authors:** Harry Hermawan, Paola Antonaci, Elke Gruyaert

**Affiliations:** 1 Department of Civil and Construction Engineering, National Taiwan University of Science and Technology, No. 43, Section 4, Keelung Road, Da’an District, Taipei 106, Taiwan; 2 Department of Structural, Geotechnical and Building Engineering (DISEG), Politecnico di Torino, Corso Duca degli Abruzzi 24, 10129 Torino, Italy; paola.antonaci@polito.it; 3 KU Leuven, Department of Civil Engineering, Materials and Constructions, Ghent Campus, Gebroeders de Smetstraat 1, 9000 Ghent, Belgium; elke.gruyaert@kuleuven.be

**Keywords:** tubular macrocapsules, self-healing concrete, mechanical properties, mix design, inert structure

## Abstract

This paper aims to assess the influence of cementitious capsules on the hardened properties of concrete, considering several parameters such as the fine fraction (*n*) of aggregates, capsule size, and capsule dosage. The presence of capsules has been formerly found to disturb packing, which eventually escalates the voids ratio of the inert skeleton. In order to understand the behavior of capsules in various packing structures, two mix design programs were developed, resulting in twenty-three concrete mixtures. The fine fraction of the aggregates was determined to be from 0.2 to 0.8. Both long and short cementitious capsules were used, with dosages of 1 to 7 vol.%. The results show that the incorporation of capsules reduced the compressive strength of concrete, and this reduction varied depending on the fine fraction, capsule dosage, and capsule size. Nevertheless, the optimum fine fraction was found to be 0.4, corresponding to the highest strength and the lowest voids ratio of the aggregate mixtures. In addition, a good bond between the capsule shell and the concrete matrix was showcased, and the embedded capsules broke during compression.

## 1. Introduction

In recent years, research has increasingly focused on the integration of innovative materials and technologies to enhance the performance of concrete, and in particular, to address the challenges posed by cracking. Cracks can compromise the structural integrity of concrete, leading to costly repairs, a reduced service life, and increased maintenance demands. As the age of current infrastructure advances and environmental challenges intensify, the need for innovative solutions to enhance the longevity and resilience of concrete structures has become increasingly pressing. Recent advancements in concrete technology have prompted the development of self-healing concrete—a transformative approach that allows concrete to autonomously repair cracks as they occur, thereby mitigating the detrimental effects of deterioration. Bacterial agents [1,2,3], crystalline admixtures [4,5,6], encapsulated materials [7,8,9], hydrogels [10,11,12], and superabsorbent polymers [13,14,15] are some of the renowned healing agents that have been widely used for the development of self-healing concrete. Each of these agents has its own mechanisms and effectiveness and is often chosen based on specific application and environmental conditions.

Among the various strategies that have previously been explored, the incorporation of capsule-like materials stands out for its effectiveness and enhanced preservation of healing agents inside the cementitious composite. These capsules are specifically designed to break upon crack formation and release their content to initiate an autonomous healing/sealing process. This method not only addresses the immediate need for repairs but also extends the life cycle of concrete structures by reducing the need for resource-intensive maintenance interventions. The design and optimization of these capsules are critical to the success of this approach [16]. Factors such as the capsule size, the wall thickness, and the type of healing/sealing agent influence not only the release mechanism but also the overall mechanical performance of the intact concrete. It is essential to achieve a balance between the functionality of the capsules and the engineering properties of the concrete matrix to ensure that the incorporation of these self-healing systems does not compromise the material’s strength and durability. Macrocapsules are considered to be more effective than microcapsules because they can store larger amounts of healing agents than micro-sized capsules.

In fact, the choice of material for the capsules depends on factors such as the desired release mechanism, its compatibility with the concrete matrix, the healing agent, the cost, and environmental considerations. Each material presents its own advantages and challenges in terms of its mechanical properties, degradation rates, and interactions with the healing agents and concrete environment. Araújo et al. [17] incorporated acrylic (PMMA) capsules and glass capsules into large concrete beams with a random distribution of the capsules. They reported that the glass capsules showed a better distribution than the PMMA capsules and that they were able to rupture when a crack of 30 μm in width occurred, while the PMMA capsules fractured at 113 μm. Formia et al. [18] examined the mechanical performance of self-compacting concrete with the addition of tubular cementitious capsules at a dosage of 1.6% of the concrete volume. The capsules were manually placed inside cube molds in three different orientations: horizontal, vertical, and random orientations. The results showed that the random distribution of capsules caused a strength reduction of 8.5%, while the other capsule orientations did not considerably influence the compressive strength. On the other hand, Tsangouri et al. [19] assessed the contribution of tubular capsules to the toughness of concrete and they found that capsules acted as a local reinforcement to the concrete, thus enhancing its resistance to damage. Based on capsule modeling by Fang et al. [20], the healing efficiency of capsules is greatly affected by the aspect ratio, for example, whether they are spherical or tubular (cylindrical).

In addition to tubular capsules, Sinha et al. [21] developed spherical and elliptical lignin-based capsules that were manufactured via 3D printing and were filled with sodium silicate as the healing agent. Gravel-sized lignin capsules with a diameter between 9.5 and 14.25 mm and a shell thickness of 0.4 mm were fabricated in accordance with the nominal size of the gravel usually used in concrete. The capsules were added during mixing to obtain a random capsule distribution. The results showed that the addition of 5 vol.% lignin capsules significantly reduced the compressive strength and splitting tensile strength by 48% and 20%, respectively, as compared with the reference concrete. The reasons for this strength reduction were (i) the high capsule volume fraction, (ii) the degradation of the lignin capsules which created weak zones inside the concrete matrix, and (iii) the capsules were lighter than the regular gravels and tended to move up during casting, resulting in a less homogeneous distribution. In another study [22] by the same authors, they modified the capsule material from lignin to polylactic acid (PLA). The diameter of the PLA capsules was slightly larger (9.5–19.05 mm) than that of the lignin capsules, and 5 vol.% PLA capsules were also added to the concrete. At 28 days, the capsule-based concrete experienced an 18.5% reduction in compressive strength and the authors suggested that the capsules acted as large inclusions within the concrete matrix, creating potential defects that contributed to stress concentration under loading.

Furthermore, in our earlier work [23], we confirmed that the presence of macrocapsules within the inert skeleton of concrete led to an increase in the voids ratio, while the physical interaction between aggregates and capsules caused a packing disturbance. It is known that the performance of concrete is considerably influenced by its composition, particularly the type and quantity of aggregates used. Aggregates, which typically comprise 60–75% of the concrete volume, play a crucial role in determining the mechanical properties, durability, and workability of the final product. The size, shape, and distribution of aggregates affect the strength of concrete, shrinkage, and resistance to cracking. For example, using well-graded aggregates with a lower voids ratio can enhance packing density, thereby improving overall concrete strength. Conversely, poorly graded aggregates with a high voids ratio can result in weaker concrete, exacerbating crack formation and hindering the self-healing capabilities of embedded macrocapsules. Therefore, achieving optimal packing between capsules and aggregates is crucial for designing effective capsule-based concrete mixtures. Since the presence of capsules increases the voids ratio of aggregates, the balance between fine and coarse aggregates must be carefully adjusted. At the time this paper was written, there were almost no studies discussing capsule–aggregate packing.

Consequently, building upon our previous study on capsule packing [23], this research further explores the parameter of voids ratio in proportioning various concrete mix designs and also examines which capsule size, capsule dosage, and aggregate composition (or fine fraction) influence the hardened properties of concrete. By optimizing the voids ratio through careful selection and grading of aggregates, concrete mixtures can be designed to not only enhance structural integrity but also maximize the effectiveness of embedded macrocapsules. Additionally, the strategic placement of capsules within the concrete matrix is essential to ensure their proper distribution, allowing them to respond effectively when cracks occur. As a note, this paper is a fragment of research from the doctoral dissertation of Harry Hermawan [24].

## 2. Voids Ratio of Aggregate–Capsule Mixture

One important factor that has gained significant attention in designing concrete mixtures is the voids ratio of aggregates, which can have a profound impact on the behavior of material. The voids ratio (*U*), defined as the ratio of the volume of voids to the volume of solid particles within the aggregate, serves as a key indicator of aggregate packing efficiency. J.D. Dewar (1999) [25] introduced the term *U* in the development of the particle packing model (PPM), which proved to be an effective tool for optimizing concrete mix designs. Nevertheless, while this model applies to basic concrete components, the addition of novel materials such as capsules necessitates experimental investigations to refine the voids ratio formulation. The novel study on the packing between capsules and aggregates was conducted by the authors in our previous publication [23]. This research evaluated changes in the voids ratio of aggregate mixtures following the introduction of macrocapsules, considering variables such as capsule dosage, capsule length, and capsule outer diameter. The assessment utilized cementitious capsules of two sizes: CEM54 capsules (long capsules, 54 mm in length, and 9 mm in outer diameter) and CEM23 capsules (short capsules, 23 mm in length, and 15 mm in outer diameter). Three types of aggregates, namely sea sand 0/2.5, gravel 4/8, and gravel 8/16, were used to create ternary blended aggregate mixtures (TAMs).

**Figure 1:**
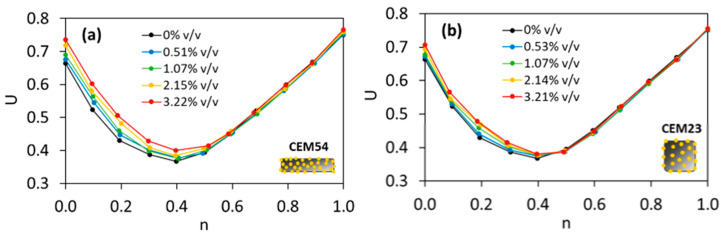
Voids ratio (*U*) of ternary aggregate mixture (TAM) with the addition of (**a**) CEM54 capsules and (**b**) CEM23 capsules in a volumetric percentage (% *v*/*v*) at different fine fractions (*n*) [23].

Following the experimental tests via the loose bulk density method (in accordance with EN 1097-3 [26]), the optimal TAM was achieved at the lowest voids ratio when the fine fraction (*n*) was 0.4. This corresponded to a composition of 40% fine aggregate (sand 0/2.5) and 60% coarse aggregate (gravel 4/8 + gravel 8/16). The coarse aggregate composition was previously fixed at *n* of 0.65 (65% gravel 4/8 + 35% gravel 8/16) based on the lowest voids ratio for coarse particles, as discussed in [23]. Thus, in this case study, the optimal inert structure consisted of 40% sand 0/4, 39% gravel 4/8, and 21% gravel 8/16. When capsules were introduced into the aggregate mixtures at dosages ranging from 0.51% to 3.22% *v*/*v*, the voids ratio was altered only at specific fine fractions, as shown in Figure 1. For CEM54 capsules, an increase in *U* was observed in aggregate mixtures with *n* below 0.6, whereas the voids ratio of aggregate–capsule mixtures remained stable for *n* ≥ 0.6 (see Figure 1a). A similar trend was observed with CEM23 capsules (see Figure 1b). This finding suggests that packing disturbance is more pronounced in mixtures with a higher content of coarse aggregate. Furthermore, the difference in *U* between CEM54 and CEM23 capsules highlights an interesting point: long capsules (Figure 1a) appear to have a greater impact on packing disturbance than short capsules (Figure 1b).

The distinct packing behaviors observed with the addition of macrocapsules motivate the authors to further investigate their effect on the hardened properties of concrete across various mix designs with different packing systems. Therefore, the main objectives of this paper are: (i) to understand the role of the voids ratio (from the inert structure) in determining the performance of capsule-based concrete, (ii) to assess the effects of capsules in relation to their dosage and size, and (iii) to study the rupturability of capsules during mechanical testing as well as the bond between the capsules and the concrete matrix. The same materials from the previous research (i.e., aggregates and cementitious capsules) [23] were used in this study.

## 3. Materials and Methods

### 3.1. Raw Material

**Figure 2:**
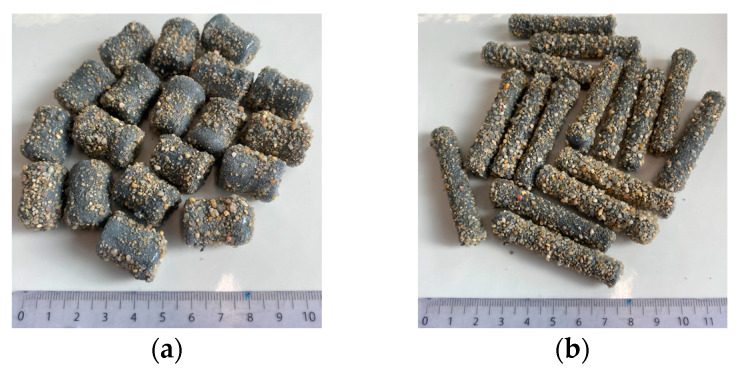
(**a**) Short capsules (CEM23) and (**b**) long capsules (CEM54).

In this study, cementitious capsules were used considering their compatibility with the concrete environment. The detailed composition and the manufacturing process of these capsules can be found in [27]. Two sizes of cementitious capsules were used: short capsules (CEM23), with a length of 23 mm and a diameter of 15 mm, and long capsules (CEM54), with a length of 54 mm and a diameter of 9 mm (see Figure 2). CEM23 capsules were designed to mimic the size of coarse aggregate, allowing them to be incorporated into the concrete mix as aggregate replacements. In contrast, CEM54 capsules were considered tubular materials, commonly used in self-healing applications, with a typical length of 50 mm. The tubular geometry was opted to store a sufficient volume of healing agent while also ensuring more efficient capsule breakage during cracking. To enhance friction and bonding with the concrete matrix, the outer surface of the capsules was coated with an epoxy layer and a sand layer. In addition, the epoxy coating provided protection for the healing agent against the harsh environment of the concrete. Each capsule contained approximately 1 mL of a water-repellent agent (WRA, Sikagard 705L, Sika AG, Baar, Switzerland), which acted as a sealing agent. The WRA was a one-component, solvent-free, silane-based water repellent with a viscosity of 9 mm^2^/s and a density of 0.9 kg/L. This agent was originally used as a water-repellent penetrating sealer for concrete flooring and has been recently used for self-healing concrete applications. The cementitious capsules were able to break when cracks reached a width of 135–240 µm, contributing to mechanical strength recovery after healing. Due to the effect of polyurethane as a healing agent, the maximum load recovery index exceeded 40% compared to the reference specimen [27]. Compared to other types of capsules (e.g., glass), cementitious capsules offer several advantages, including reduced brittleness, lower risk of alkali–silica reaction, and greater compatibility with the surrounding concrete matrix [24,27].

CEM III/A 42.5N (CBR, Ghent, Belgium), consisting of 52% clinker and 48% blast furnace slag, was used as the binder in the concrete. Sea sand 0/2.5 was used as the fine aggregate, while two fractions of gravel (4/8 and 8/16) served as the coarse aggregates. The specific gravities of sand 0/2.5, gravel 4/8, and gravel 8/16 were 2.67, 2.59, and 2.60, respectively. A polycarboxylate-ether (PCE) superplasticizer (Fluvicon 801, CUGLA B.V., Breda, The Netherlands) with a 20% solid content was used to improve the workability of the fresh mixture.

### 3.2. Concrete Mix Design

**Figure 3:**
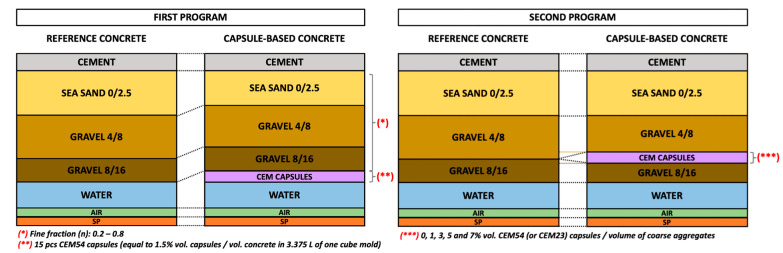
Schematic illustration of concrete mix design modifications for the introduction of macrocapsules.

Two programs of mix design modifications (C30/37 type) were studied to evaluate the effects of macrocapsules on packing. An illustration of these two programs of capsule-based concrete mix design is depicted in Figure 3.

The first program aimed to investigate the effect of capsules in various concrete mix designs, focusing on the voids ratio of capsule–aggregate mixtures. Our previous study [23] (refer to Section 2) indicated that the lowest voids ratio was achieved in a ternary aggregate mixture (TAM) with *n* of 0.40 (40% sand 0/2.5; 60% (gravel 4/8 + gravel 8/16)). Moreover, the packing disturbance within the inert skeleton occurred only when the fine fraction (*n*) was below a certain threshold. Lower fine fractions led to greater disturbance caused by the capsules compared to higher fine fractions. To test this, the aggregate proportion was taken as the main variable, with the fine fraction ranging from 0.2 to 0.8 (see Table 1). While some of these mixtures may not be practical for real-world applications, they were included to assess the impact of capsules on different packing structures. The capsule dosage was fixed at 1.5% v_caps_/v_con_, based on the limited availability of cementitious capsules and typical dosages used in past studies (0.7–2.7% v_caps_/v_con_) [24]. For example, the notation “1.5CEM54-30” represents a 1.5% capsule dosage, long cementitious capsules (CEM54), and a fine fraction of 30% (or 0.3). The first seven mixtures, which contained no capsules, served as reference samples (i.e., 0CEM54-20, …, 0CEM54-80). The capsule dosage (% v_caps_/v_con_) refers to the volume of capsules relative to the total volume of concrete. For a 1.5% v_caps_/v_con_, 0.051 L of capsules (15 pcs) was required per cube mold, with three cube specimens prepared for each mixture. The mix designs are presented in Table 1. Cement content, *w*/*c*, and SP dosage remained constant for both reference and capsule-based mixtures. The amount of aggregates in capsule-based mixtures was slightly adjusted to compensate for the volume of capsules, ensuring a consistent 1 m^3^ volumetric design across all mixtures.

The second program aimed to assess the impact of capsule dosage on the hardened concrete properties. As shown in [23], the presence of capsules did not affect the packing of fine aggregates; however, increasing the capsule dosage raised the voids ratio of coarse aggregates. To minimize the voids ratio, capsules were incorporated as replacements for coarse aggregates in the mixture. Therefore, the capsule dosage was expressed as the volume of capsules relative to the volume of coarse aggregates (v_caps_/v_agg_). The reference mixture, 0CEM54-40, was selected due to its low voids ratio. Both long (CEM54) and short (CEM23) capsules were used at dosages of 1, 3, 5, and 7% v_caps_/v_agg_, based on the limited availability of cementitious capsules and typical ranges from past studies (approximately 1.6–6.3% v_caps_/v_agg_) [24]. Concrete mixtures were cast in 150 mm cube molds (3.375 L per mold). In the reference mix, 41.6% of the volume consisted of coarse aggregates, or approximately 1.40 L per cube. Capsules replaced a portion of the coarse aggregates, with different quantities used for long and short capsules. For dosages of 1, 3, 5, and 7% v_caps_/v_agg_, 5, 13, 21, and 29 long capsules (CEM54) and 4, 11, 18, and 25 short capsules (CEM23) were used per cube, respectively. Three cube specimens were cast for each mixture, and the mix designs are listed in Table 2. Cement content, sand content, *w*/*c*, and SP dosage remained constant for both reference and capsule-based mixtures. In capsule-based mixtures, the amount of coarse aggregate was adjusted to maintain a consistent 1 m^3^ volumetric design for all concrete mixtures.

### 3.3. Mixing and Casting Process

**Figure 4:**
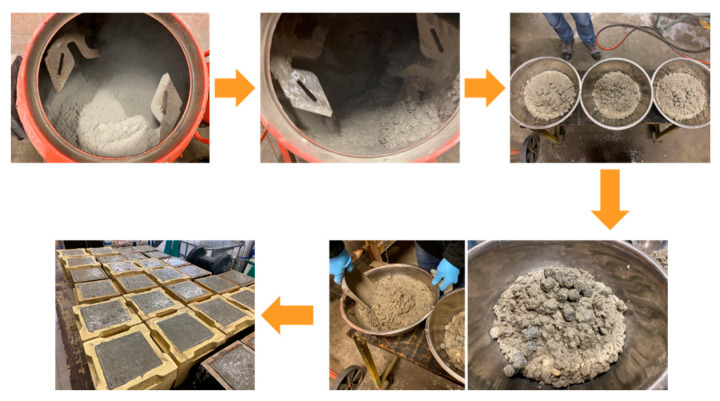
Procedure of casting the capsule-based concrete.

A target volume of 20 L of concrete was prepared for each mixture to assess the hardened properties. The mixing process began with all dry materials being mixed for 30 s in a drum mixer (Lescha SM 145 S, ALTRAD Lescha Atika GmbH, Burgau, Germany). Next, the mixing water was added, followed by 2 min of mixing before introducing the superplasticizer. The mixer continued running for a total of 5 min, after which the fresh mix was taken out. The fresh mixture was then divided into three bowls, each with a comparable volume of concrete. Depending on the desired capsule dosage, the appropriate number of capsules was added to each bowl and mixed manually until a homogeneous distribution was achieved. The mixture was subsequently poured into three 150 mm cube molds, ensuring the same number of capsules on each cube. In this way, a random distribution of capsules was attained. To ensure proper consolidation, the fresh mixture was compacted using a vibrating table. The reference mixtures (without capsules) were cast following the same procedure as the capsule-based mixtures. After 24 h, the specimens were demolded and placed in a water tank at 20 °C for curing. The process of casting capsule-based concrete is illustrated in Figure 4.

### 3.4. Testing Program

All specimens from the twenty-three mixtures were removed from the curing tank at 28 days. The ultrasonic pulse velocity (UPV) test, based on EN 12504-4 [28], was first performed on the hardened cube specimens to assess their compactness with and without capsules. During the UPV test, Pundit transducers (Proceq, Schwerzenbach, Switzerland) were placed at the center of two parallel faces of each cube, and the test was conducted in direct transmission mode. The tests were repeated by moving the transducers to the other faces of each cube, resulting in two UPV measurements per specimen (on molding surfaces). The average velocity was then recorded for each specimen. Additionally, hardened densities were measured, and compression tests were conducted in accordance with EN 12390-3 [29]. A qualitative evaluation of capsule breakage and the bond between the capsules and the concrete matrix was also performed as detailed in Section 4.4.

An additional study was conducted to evaluate the resistance of cementitious capsules during the mixing process. A drum mixer (Lescha SM 145 S, ALTRAD Lescha Atika GmbH, Burgau, Germany) was used with 20 L of the reference mixture (REF or 0CEM54-40), incorporating 20, 40, and 80 capsules for both CEM23 and CEM54 types. The mixing procedure followed the same steps as outlined in Section 3.3, with one exception that, after 5 min of mixing, the capsules were added and mixed for an additional 3 min to ensure uniform distribution. To assess capsule survivability, the fresh concrete was placed on a sieve and submerged in water. The sieve was then shaken manually until the aggregates and capsules were separated from the fresh mortar. This allowed for the collection of capsules after mixing, enabling the calculation of their survivability, defined as the total number of intact capsules over the total number of capsules initially added.

## 4. Result and Discussion

### 4.1. The Effect of Fine Fraction in Capsule-Based Concrete Mixtures (In Relation to the First Program)

**Figure 5:**
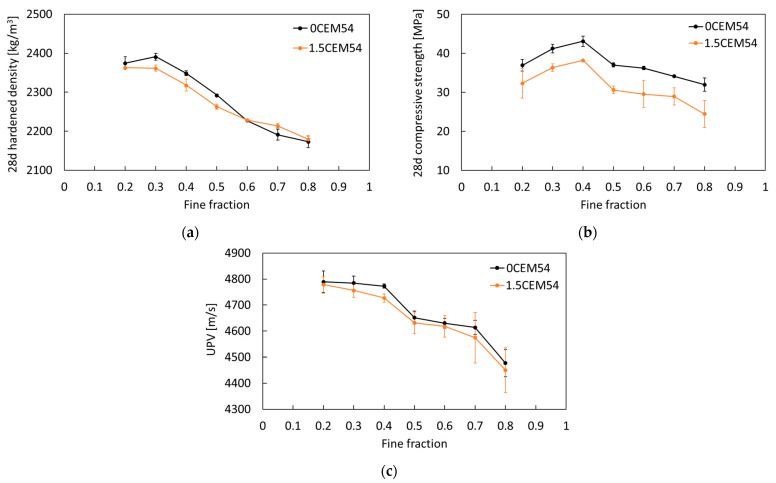
Results of (**a**) hardened density, (**b**) compressive strength, and (**c**) UPV of reference and capsule-based concretes tested at 28 days from the first program.

The hardened properties of both reference and capsule-based concretes were evaluated at 28 days. The results for hardened density and compressive strength are presented in Figure 5a and Figure 5b, respectively. Results showed that there was no considerable difference in hardened densities between reference and capsule-based concretes. Nevertheless, the addition of 1.5 vol.% macrocapsules led to a reduction in compressive strength across all mix designs. The extent of this strength reduction varied depending on the fine fraction (*n*). Specifically, for *n* of 0.2–0.4, the strength reduction was approximately 11–12% compared to the corresponding reference concretes. For *n* of 0.5–0.7, the reduction increased to 15–18%, while for *n* of 0.8, the reduction reached 23%, the highest among all fine fractions. These results highlight that the effect of capsules on compressive strength depends on the mix design. Two possible explanations for this trend are: (1) the random distribution of capsules across all mixtures results in varying effects on concrete strength, and (2) differences in aggregate contribute to the strength reduction when capsules are introduced into the matrix. Comparing all fine fractions, the mixture with *n* of 0.4 achieved the highest compressive strength, both with and without capsules. This aligns with the findings of our previous study [23], which identified *n* of 0.4 as the optimal fine fraction for achieving the lowest voids ratio in the aggregate mixture.

In addition, the UPV results are summarized in Figure 5c and a statistical analysis using one-way ANOVA was conducted on the UPV values, with the result presented in Table 3. In general, only a minor reduction in matrix compactness was observed following the inclusion of macrocapsules. It was found that the addition of 1.5 vol.% capsules reduced the velocity in a range of 0.2–1.0% for *n* between 0.2 and 0.8, compared to the reference mixtures. All in all, the incorporation of macrocapsules mainly affects the compressive strength of concrete, while having no considerable effects on hardened density and the compactness.

**Figure 6:**
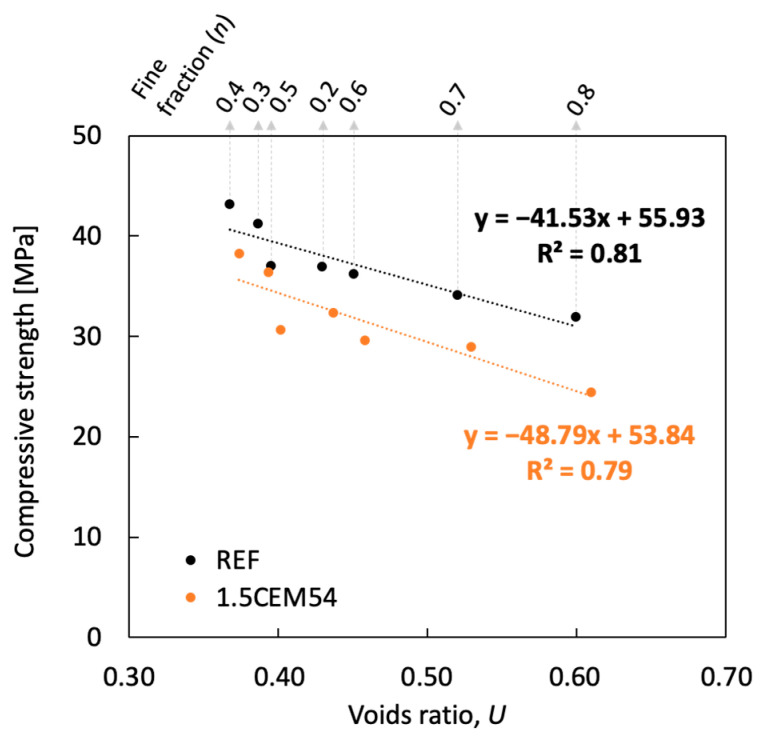
Relationship between the compressive strength and the voids ratio of aggregates.

A relationship between compressive strength and the voids ratio of the aggregate mixture used in the mix design is constructed in Figure 6. A good coefficient of determination (*R*^2^) was obtained, demonstrating the ability to predict the compressive strength of both reference and capsule-based concrete as a function of the voids ratio. It should be noted that only one capsule dosage (1.5 vol.%) was studied in this program to evaluate the effect of capsules across different mix designs. As shown in Figure 6, an increase in the voids ratio of the inert skeleton (capsule–aggregate mixture) led to a decrease in compressive strength. This reduction occurs because the large spacing in the inert skeleton that has to be filled by the cement paste. The presence of capsules generates additional voids due to their geometric differences and interactions with coarse aggregates. In addition, since the capsules have lower strength compared to coarse aggregates, their inclusion—whether as an additive or a partial replacement for stone—inevitably contributes to a decline in concrete strength. Further research is needed to refine models predicting compressive strength in relation to capsule dosage across different mix designs.

### 4.2. The Effect of Capsule Size and Capsule Dosage in Capsule-Based Concrete Mixtures (In Relation to the Second Program)

**Figure 7:**
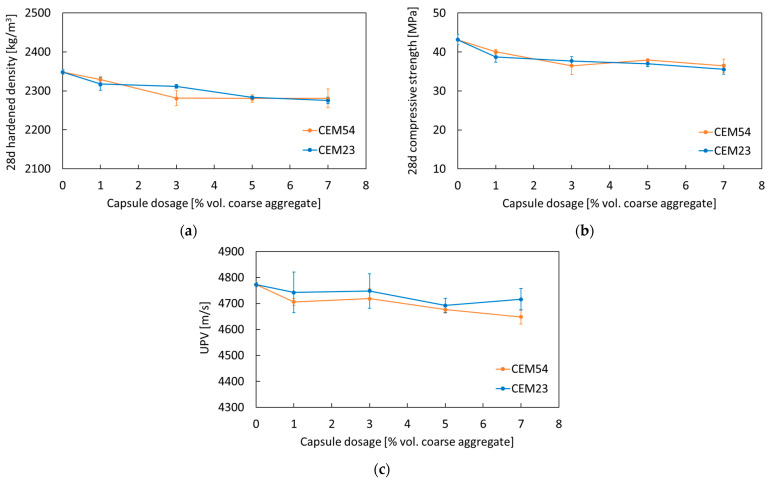
Results of (**a**) hardened density, (**b**) compressive strength, and (**c**) UPV of reference and capsule-based concretes tested at 28 days from the second program.

From Figure 7a, the hardened density of concrete slightly decreased by 1–3% following the addition of capsules at dosages ranging from 1 to 7 vol.%. No considerable difference in hardened density was observed between long and short capsules. The compressive strength of capsule-based concrete was mainly evaluated and the results are presented in Figure 7b. When the capsule dosage was set at 1, 3, 5, and 7 vol.%, the strength reduction was recorded at 7, 15, 12, and 15% (with the use of CEM54 capsules), respectively, and at 10, 13, 14, and 18% (with the use of CEM23 capsules), respectively, in comparison to the reference concrete. Three possible reasons for the decline in compressive strength can be identified: (1) capsules act as “weak” spots within the concrete matrix, (2) capsules occupy a portion of the concrete volume, and (3) capsules disturb the packing structure.

The compactness of concrete, as assessed by the UPV test, showed a slight reduction after the incorporation of capsules (see Figure 7c). The ultrasonic velocity decreased by less than 3% with the addition of 1–7 vol.% capsules (either CEM23 or CEM54). In absolute terms, CEM23 capsules appeared to result in slightly better compactness compared to CEM54 capsules. This may be attributed to their physical form where CEM23 capsules were designed to resemble coarse aggregates, allowing them to blend more effectively within the mix. In contrast, CEM54 capsules, being long and tubular, may induce a greater impact on the packing structure (see Figure 1), potentially creating more disruptions. Nevertheless, the differences in ultrasonic velocities between these capsules were not statistically significant, as confirmed by the analysis of variance (ANOVA), where *p*-values were greater than 0.05 (see Table 3).

### 4.3. Capsule Breakage During Compression Tests

**Figure 8:**
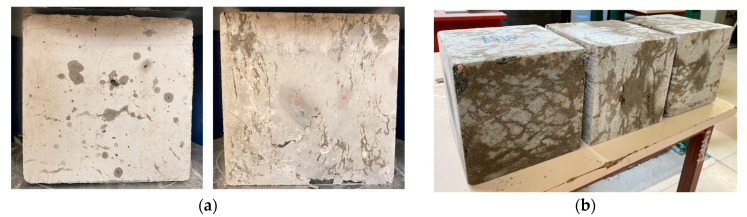
(**a**) Wet spots as an indication of capsule breakage during compression tests, (**b**) the spread of WRA via microcracks and capillary action.

During the compression tests, “wet” spots were observed on the concrete surface, as shown in Figure 8. This indicates that some capsules inside the concrete ruptured under high loading, releasing WRA through the cracks. While it was difficult to confirm the exact number of broken capsules, the results suggest that at least some capsules were successfully activated upon cracking. A higher number of capsules could potentially increase the likelihood of capsule breakage. Figure 8 shows the typical spreading of WRA after compression, with crazing cracks on the surface being sealed as a result of the released agent. In this case, WRA was absorbed by the concrete matrix through cracks and capillary action, demonstrating its self-sealing effect.

**Figure 9:**
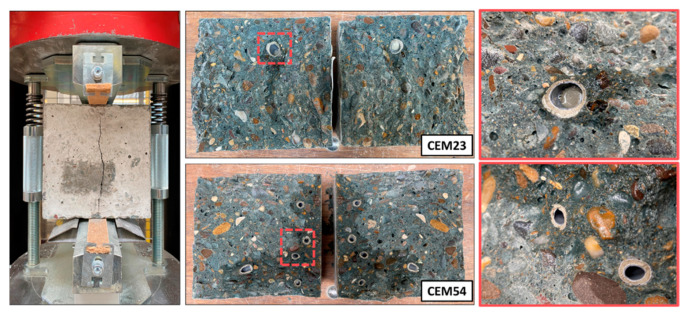
Observation of the broken capsules in a certain crack plane after splitting cubes (note: the darkest concrete surface is the area covered with sealing agent following the capsule breakage).

After subjecting all specimens from the second program (e.g., 1CEM54, …, 7CEM23) to compression tests, they were split until failure using the tensile splitting test to count the number of broken capsules along the crack plane. As shown in Figure 9, the split was intentionally made near the center of the cube. Once the cube was split, the number of ruptured capsules was recorded. Table 4 summarizes the total number of embedded capsules and those that ruptured within the crack plane, based on three repetitions per mixture. Among the CEM54 mixtures, 7CEM54 exhibited the highest number of broken capsules, likely due to its high capsule dosage. Conversely, mixtures with lower capsule dosages showed fewer broken capsules. For CEM23 mixtures, capsule breakage was considerably lower compared to CEM54 mixtures, which may be attributed to their shape. The CEM23 capsules, being similar in size to coarse aggregates, were more affected by their positioning within the matrix and the crack itself. In contrast, CEM54 capsules, being longer, had a higher likelihood of being intersected by cracks, increasing the probability of rupture when cracking occurred.

### 4.4. Bond Between the Capsule Shell and the Concrete Matrix

**Figure 10:**
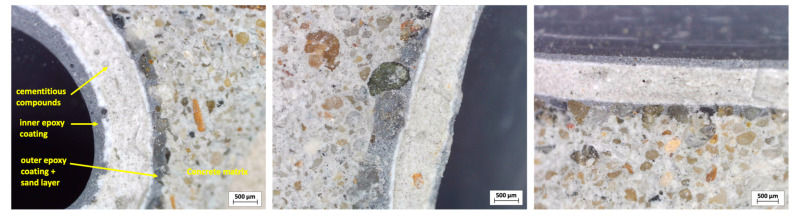
Bond observation between the capsule shell (CEM54) and the concrete matrix.

The broken capsules from the split cubes were observed using an optical microscope (TOOLCRAFT USB microscope 5MP, Conrad Electronic SE, Hirschau, Germany) at the interface between the capsule shell (CEM54, as an example) and the concrete matrix, as depicted in Figure 10. The microscopy observations confirmed that the capsules were well attached to the concrete matrix. Notably, no delamination was observed at the capsule–matrix interface, even after the specimen had cracked.

**Figure 11:**
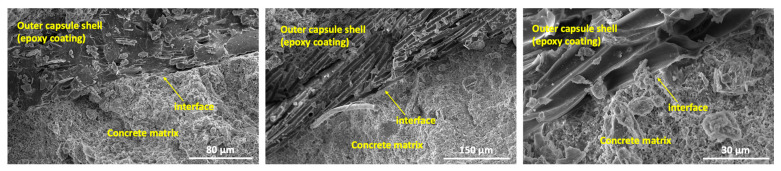
Microstructure images of the interface between the outer capsule shell and the concrete matrix.

**Figure 12:**
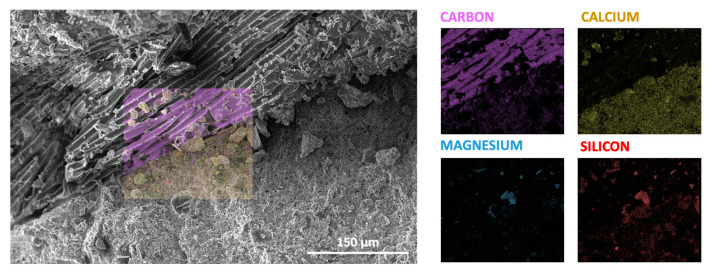
EDX analysis of concrete matrix–outer capsule shell interface.

To further verify the bond between the capsules and the concrete matrix, scanning electron microscopy (SEM) (PhenomXL, Thermo Fisher Science, Waltham, MA, USA) was performed on a capsule–matrix fragment. Before imaging, the sample was coated with gold, and microstructure images were obtained using a secondary electron detector (SED), as presented in Figure 11. The SEM investigation confirmed that the capsule shell remained well attached to the concrete matrix without signs of delamination. Additionally, energy dispersive X-ray (EDX) spectroscopy analysis (Figure 12) provided further confirmation of the material composition. The outer capsule shell was found to be rich in carbon, which corresponds to the epoxy coating, while the concrete matrix itself was rich in calcium, distinguishing the two materials.

### 4.5. Capsule Resistance Towards Concrete Mixing

**Figure 13:**
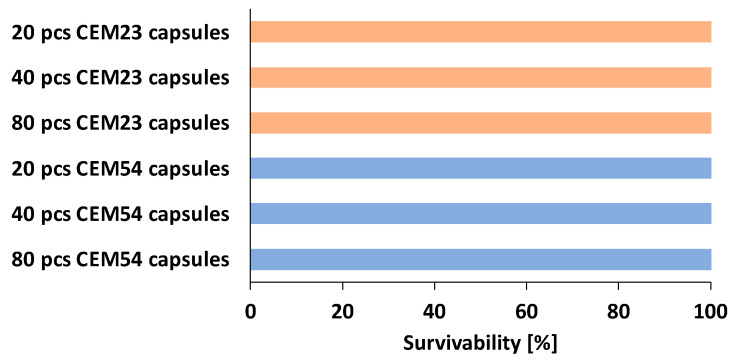
Capsule survivability (based on a single test).

Figure 13 presents the percentage of capsules that survived after being mixed with the concrete components in the mixer. Interestingly, both short and long capsules remained fully intact, with no significant damage to their capsule shells. The survivability of long capsules (CEM54) when mixed in two different types of mixers (a drum mixer and a planetary mixer) was previously assessed in [24]. The results showed that 100% survivability was achieved when capsules were mixed in a drum mixer, while 70–95% survivability was recorded in a planetary mixer. This proof-of-concept test confirms that the cementitious capsules are sufficiently robust to withstand the mixing forces encountered during concrete preparation.

### 4.6. Practical Application of Capsules in Large-Scale Concrete Production

Capsules are incorporated into concrete in research studies using two primary methods: (i) manual placement and (ii) direct addition. The most common method is manually placing the capsules within the cover zone of the mortar or concrete. This is typically performed by installing a network of wires inside the specimen mold to which the capsules are attached with glue. However, this method may be impractical for large-scale applications, as manually installing capsules one by one in a large concrete structure can be cumbersome. For example, Van Tittelboom et al. [30] manually placed 350 glass capsules with the help of wires and glue in the cover zone of a concrete beam, while Siahkouhi et al. [31] incorporated over 800 glass capsules into a concrete railway sleeper through manual installation. While this method may be feasible for lab-scale applications, directly adding capsules during mixing offers significant advantages in terms of time efficiency and practicality. The downsides of this approach are that capsules are randomly distributed and the capsule breakage will certainly depend on the location of capsules. However, as long as they are well distributed, they can effectively promote local repair over a sufficiently large area. Araújo et al. [17] demonstrated the feasibility of mixing and placing 3250 capsules in 150 L of concrete with a random distribution. Thus, direct addition is a more practical and scalable approach than manual placement.

Additionally, the design of capsules is crucial; they must be robust enough to withstand mixing forces but brittle enough to break when cracks form, ensuring capsules serve their self-healing function effectively, as showcased in this study. Compared to other self-healing materials (e.g., bacteria, crystalline admixtures, and polymers), capsules can become a more cost-effective option when produced on a larger scale, as they can be manufactured by cutting readily available tubular materials (e.g., glass tube and acrylic tube) or through extrusion or by means of other manufacturing processes. However, the production and handling of capsules can be time-consuming, as the process involves several steps from the tube manufacturing and the injection of healing agents to sealing the capsule ends. Consequently, further research is required to identify the most efficient method for capsule production and determine whether capsules offer a more economical solution compared to other self-healing systems.

## 5. Conclusions

This study aims to evaluate the effect of tubular macrocapsules in various concrete mix designs, considering their inert skeleton. Two types of cementitious capsules were used: CEM54 (54 mm in length and 9 mm in outer diameter) as long capsules and CEM23 (23 mm in length and 15 mm in outer diameter) as short capsules. The modification of mix designs was divided into two programs: (1) the first program involved the incorporation of 1.5 vol.% CEM54 capsules in seven concrete mix designs with different fine fractions (*n*, the volumetric ratio of sand to the total volume of all aggregates) ranging from 0.2 to 0.8; and (2) the second program examined the use of capsules as a replacement for coarse aggregates, evaluating the effect of capsule dosage from 1 to 7 vol.%. The main findings are presented as follows:

1. The incorporation of 1.5 vol.% capsules led to a decrease in compressive strength. When the *n* was set from 0.2–0.4, a strength reduction of 11–12% was observed, while for *n* of 0.5–0.8, the reduction increased to 15–23%. Additionally, increasing the capsule dosage from 1 to 7 vol.% resulted in a gradual decline in compressive strength.
2. Long capsules had a greater impact on concrete strength than short capsules, due to their shape and physical interaction with concrete components.
3. The highest compressive strength was achieved in the concrete mixture with *n* of 0.4, which aligns well with the lowest voids ratio observed in the ternary aggregate mixture (TAM). A good relationship between the compressive strength and the voids ratio of the TAM was established, where a higher voids ratio of the TAM led to a lower compressive strength.
4. The addition of capsules had no considerable effects on the hardened density or the compactness of concrete, as confirmed by UPV tests.
5. During compression tests, some capsules ruptured, as indicated by wet spots on the concrete surface. The released sealing agent was able to widely spread throughout the matrix via cracks and capillary action.
6. Microscopic and microstructure observations confirmed a good bond between the capsule shell and the concrete matrix, with no signs of delamination.

Following this study, further research is necessary to understand the durability of capsule-based concrete and the behavior of larger concrete elements subjected to more complex loads than the one presented in this paper.

## Figures and Tables

**Table 1:** Mix designs of capsule-based concrete from the first program (% v_caps_/v_con_ = volume of capsules/volume of concrete).

**Material**	**Unit**	**0CEM54-20**	**0CEM54-30**	**0CEM54-40**	**0CEM54-50**	**0CEM54-60**	**0CEM54-70**	**0CEM54-80**
CEM III/A	kg/m^3^	325	325	325	325	325	325	325
Sea sand 0/2.5	kg/m^3^	370	555	740	924	1109	1293	1477
Gravel 4/8	kg/m^3^	937	820	701	585	468	351	234
Gravel 8/16	kg/m^3^	504	441	378	315	252	189	126
Effective water	kg/m^3^	163	163	163	163	163	163	163
Superplasticizer	kg/m^3^	0.00	0.00	0.89	1.00	1.20	1.50	1.80
Effective *w*/*c*	-	0.50	0.50	0.50	0.50	0.50	0.50	0.50
CEM54 capsules	% v_caps_/v_con_	-	-	-	-	-	-	-
Fine fraction	-	0.2	0.3	0.4	0.5	0.6	0.7	0.8
**Material**	**Unit**	**1.5CEM54-20**	**1.5CEM54-30**	**1.5CEM54-40**	**1.5CEM54-50**	**1.5CEM54-60**	**1.5CEM54-70**	**1.5CEM54-80**
CEM III/A	kg/m^3^	325	325	325	325	325	325	325
Sea sand 0/2.5	kg/m^3^	362	543	724	904	1085	1265	1445
Gravel 4/8	kg/m^3^	916	802	686	572	457	343	229
Gravel 8/16	kg/m^3^	494	432	370	308	246	185	123
Effective water	kg/m^3^	163	163	163	163	163	163	163
Superplasticizer	kg/m^3^	0.00	0.00	0.89	1.00	1.20	1.50	1.80
Effective *w*/*c*	-	0.50	0.50	0.50	0.50	0.50	0.50	0.50
CEM54 capsules	% v_caps_/v_con_	1.5	1.5	1.5	1.5	1.5	1.5	1.5
Fine fraction (*n*)	-	0.2	0.3	0.4	0.5	0.6	0.7	0.8

**Table 2:** Mix designs of capsule-based concrete from the second program (note: % v_caps_/v_agg_ = volume of capsules/volume of coarse aggregates).

**Material**	**Unit**	**REF**	**1CEM54**	**3CEM54**	**5CEM54**	**7CEM54**	**1CEM23**	**3CEM23**	**5CEM23**	**7CEM23**
CEM III/A	kg/m^3^	325	325	325	325	325	325	325	325	325
Sea sand 0/2.5	kg/m^3^	740	740	740	740	740	740	740	740	740
Gravel 4/8	kg/m^3^	701	694	680	666	652	694	680	666	652
Gravel 8/16	kg/m^3^	378	374	366	359	351	374	366	359	351
Effective water	kg/m^3^	163	163	163	163	163	163	163	163	163
Superplasticizer	kg/m^3^	0.89	0.89	0.89	0.89	0.89	0.89	0.89	0.89	0.89
Effective *w*/*c*	-	0.50	0.50	0.50	0.50	0.50	0.50	0.50	0.50	0.50
CEM54 capsules	% v_caps_/v_agg_	-	1	3	5	7	-	-	-	-
CEM23 capsules	% v_caps_/v_agg_	-	-	-	-	-	1	3	5	7
Fine fraction (*n*)	-	0.4	0.4	0.4	0.4	0.4	0.4	0.4	0.4	0.4

**Table 3:** Statistical results on the UPV values based on one-way ANOVA (note: *p*-value of 0.05 is a significance threshold).

First Program		Second Program	
Mixture Comparison	*p*-Value	Mixture Comparison	*p*-Value
0CEM54-20 vs. 1.5CEM54-20	0.754	1CEM54 vs. 1CEM23	0.471
0CEM54-30 vs. 1.5CEM54-30	0.256	3CEM54 vs. 3CEM23	0.543
0CEM54-40 vs. 1.5CEM54-40	0.012	5CEM54 vs. 5CEM23	0.420
0CEM54-50 vs. 1.5CEM54-50	0.969	7CEM54 vs. 7CEM23	0.079
0CEM54-60 vs. 1.5CEM54-60	0.890		
0CEM54-70 vs. 1.5CEM54-70	0.199		

**Table 4:** Number of broken capsules in a certain crack plane after splitting cubes.

Mixture	Number of Embedded Capsules Per Cube	Number of Broken Capsules in a Certain Crack Plane		
		Cube 1	Cube 2	Cube 3
1CEM54	5	2	0	0
3CEM54	13	1	0	0
5CEM54	21	5	4	2
7CEM54	29	5	2	7
1CEM23	4	0	0	1
3CEM23	11	0	0	0
5CEM23	18	0	0	0
7CEM23	25	0	0	2

## Data Availability

The data presented in this study are openly available on the SMARTINCS Zenodo platform.
